# Topology‐Matching Design of an Influenza‐Neutralizing Spiky Nanoparticle‐Based Inhibitor with a Dual Mode of Action

**DOI:** 10.1002/anie.202004832

**Published:** 2020-07-08

**Authors:** Chuanxiong Nie, Badri Parshad, Sumati Bhatia, Chong Cheng, Marlena Stadtmüller, Alexander Oehrl, Yannic Kerkhoff, Thorsten Wolff, Rainer Haag

**Affiliations:** ^1^ Institute of Chemistry and Biochemistry Freie Universität Berlin Takustr. 3 14195 Berlin Germany; ^2^ Unit 17 Robert Koch Institut Seestr. 10 13353 Berlin Germany; ^3^ Department of Chemical Engineering and Biotechnology University of Cambridge Cambridge CB3 0AS UK; ^4^ College of Polymer Science and Engineering Sichuan University No.24 South Section 1, Yihuan Road 610065 Chengdu China

**Keywords:** antiviral agents, inhibitors, influenza, nanoparticles, topology matching

## Abstract

In this study, we demonstrate the concept of “topology‐matching design” for virus inhibitors. With the current knowledge of influenza A virus (IAV), we designed a nanoparticle‐based inhibitor (nano‐inhibitor) that has a matched nanotopology to IAV virions and shows heteromultivalent inhibitory effects on hemagglutinin and neuraminidase. The synthesized nano‐inhibitor can neutralize the viral particle extracellularly and block its attachment and entry to the host cells. The virus replication was significantly reduced by 6 orders of magnitude in the presence of the reverse designed nano‐inhibitors. Even when used 24 hours after the infection, more than 99.999 % inhibition is still achieved, which indicates such a nano‐inhibitor might be a potent antiviral for the treatment of influenza infection.

## Graphical abstract

**Influenza meets its match**: Based on the principle of topology matching, a potent nanoparticle‐based inhibitor for influenza A was designed that shows heteromultivalent inhibition of the receptor‐binding viral proteins. This inhibitor is capable of neutralizing influenza A virus extracellularly and blocking its interaction with the host cell receptors. This principle could potentially also be applied to the inhibition of other viruses with spiky surfaces.

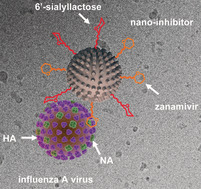

Besides the recent SARS‐CoV‐2 pandemic, outbreaks of seasonal or pandemic influenza A virus (IAV) have also challenged public health due to the high mutation rates of viral glycoprotein genes and the potential for human infection by animal strains.^1^ Due to the lack of universal influenza vaccines, a robust virus‐neutralizing therapeutic is needed.^2^ IAV is an enveloped RNA virus, the membrane of which anchors two viral proteins that regulate interactions of the virion with the host cells, hemagglutinin (HA) and neuraminidase (NA).^3^ For infection, IAV uses HA to bind to sialic acid on the host cell membrane. After completion of the replication cycle, the viral NA cleaves sialic acid from surface receptors to allow virion release, so‐called budding.^4^ Recent evidence also demonstrates that NA helps IAV penetration in the mucus by cleaving HA decoy receptors, which reveals the crucial role for a balanced HA/NA interplay for the binding behavior.^5^ Multivalent sialylated nanostructures have been developed to inhibit HA and block viral entry to host cells.^6^ However, heteromultivalent inhibitors engaging both HA and NA have been rarely been reported.^15^

From a topological viewpoint, the virion of IAV is nano‐sized particle around 100 nm with a spiky surface generated by the HA and NA.^7^ For an inhibitor, especially nanoparticle‐based inhibitors (nano‐inhibitors), matching the size and topology to the virion is essential in order to achieve robust binding to compete with the virus/cell interaction. For this purpose, flexible nanomaterials are favored to afford the viral binding ligands, but they also face the problem of overcoming the internal stress of the scaffold nanomaterials.^8^ In our new approach, we used a rigid nanoparticle with a matching nanotopology to the viral particle, which binds more strongly than the flexible nanomaterials.^9^

The aim of this study was to develop a nano‐inhibitor with the principle of topology‐matching design. The inhibitor should not only show dual inhibitory effects on HA and NA, but should also exhibit a matched nanotopology to IAV virion, which is expected to increase the contact area and enhance the binding. To achieve that, a virus‐like nanoparticle (VLNP) with nanospikes was first synthesized according to an earlier report.^10^ Except for the difference of the morphology, the VLNP was the same as its smooth control nanoparticle for size and density (Figure S2 in the Supporting Information). Then, the nano‐inhibitor was synthesized through the functionalization of the VLNP with linear polyglycerol‐sialyllactose (LPG‐SAL) and LPG‐zanamivir (LPG‐Zan) through copper‐free SPAAC click reactions as shown in Figure 1 a. Despite the different subtypes of HA, human IAV strains tend to bind the 6′‐sialyllactose for entry into host cells.^11^ In this study, 6′‐sialyllactose was conjugated onto the nanoparticles via an LPG linker in a multivalent manner as shown in Figure 1 b, since polyglycerol‐based multivalent structures have been shown to efficiently to block virus infection in our former studies.6c, 6d In order to inhibit NA, an approved NA inhibitor, zanamivir, was also conjugated to the VLNPs, similarly in a multivalent manner.^12^ The resulted heteromultivalent structure is expected to bind IAV virion strongly as shown in Figure 1 c.

**Figure 1:**
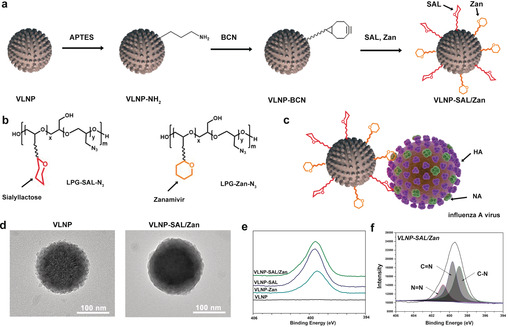
a) Synthetic outline for the topology‐matching design of nano‐inhibitors towards IAV. b) Structure of LPG‐SAL‐N_3_ and LPG‐Zan‐N_3_, which were used for the functionalization. Detailed structures are shown Figure S1. c) Proposed binding patterns between VLNP‐SAL/Zan and influenza virus particles. d) HR‐TEM images for the VLNPs before and after the functionalization. Scale bar: 100 nm. The images for smooth nanoparticle are shown in Figure S3. e) XPS N1s spectra for the VLNPs with the functionalization. f) Peak analysis for the XPS N1s spectra for VLNP‐SAL/Zan.

High‐resolution TEM (HR‐TEM) images were acquired to show the LPG‐SAL and LPG‐Zan conjugation on the VLNPs (Figure 1 d). The bare VLNPs had a rough surface with clear and sharp edges. With the conjugation of LPG‐SAL and LPG‐Zan, the spiky nanostructure is smoother, but a rough surface was still noticed, which shows advantages for the efficient binding of virus in the following studies. The chemical structure of VLNP‐SAL/Zan was studied by XPS analysis, as shown in Figure 1 e,f. From the XPS N1s scan, the emerge of the N1s signal for VLNP‐SAL/Zan indicated that the LPG‐SAL and LPG‐Zan have been conjugated to the VLNP. For the N1s scan, three characteristic peaks were detected as shown in Figure 1 e. Overall, the results of HR‐TEM and XPS revealed that the VLNPs had been successfully functionalized with LPG‐SAL and LPG‐Zan.

Binding with the virus was studied by a centrifuge western blotting as shown in Figure 2 a,b and Figure S4, and the band intensity for the viral nucleoprotein (viral NP) reveals the amounts of virus binding to the nanoparticles. For the LPG‐functionalized smooth nanoparticles (Figure S4), no binding was detected. But VLNP‐LPG showed weak binding with the virus, which is likely due to the spiky nanostructures on the surface. All the SAL nanoparticles showed robust binding to influenza virus and VLNP‐SAL/Zan was better than VLNP‐SAL. This should be attributed to the additional interactions between Zan and NA, which not only added extra binding sites to the nanoparticles, but also enhanced the SAL/HA interaction.^13^ It was also clear that the nanospikes on the surface have a positive effect on the viral binding. Cryo‐TEM image was acquired to show the effect of the nanospikes on the viral binding as shown in Figure 2 c. From the images, it can be seen that the surface proteins can insert into the gaps of the nanospikes, which increased the contact area to benefit the binding. Furthermore, aggregates of the VLNP‐SAL/Zan with the virus particles were also noticed.

**Figure 2:**
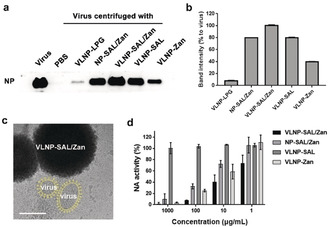
a) Western blot of influenza nucleoprotein (NP) that reveals viral binding to the nanoparticles.b) Band‐intensity analysis for the western blot in (a). c) Cryo‐EM images for virus binding to VLNP‐SAL/Zan. The virus is marked yellow for a better view. Images without marks are shown in Figure S5. Scale Bar: 100 nm. d) Inhibition of NA activity by the nano‐inhibitors. Values are expressed by mean±SD, *n*=4.

NA inhibition was studied by MU‐NANA assay (Figure 2 d). The nanoparticles with zanamivir all showed inhibition of NA and the IC_50(NA)_ values for the VLNP‐SAL/Zan, NP‐SAL/Zan, and VLNP‐Zan particles are 5.38±1.37, 40.34±10.82, 20.54±1.31 μg mL^−1^, respectively. By comparing the results for VLNP‐SAL/Zan and NP‐SAL/Zan, an enhancement by a factor of 8 was also noticed for the spiky nanostructures, which can be attributed to increased virus binding by the spiky nanostructures.

We then investigated whether the binding of IAV virions to MDCK II cells can be blocked by the nano‐inhibitors, to demonstrate that they act through a binding‐decoy mechanism (Figure 3 a–c). In this test, virions of influenza A/X31 (H3N2) were labelled with octadecyl rhodamine B chloride (R18) and incubated with the nano‐inhibitors for 45 minutes.^14^ After being incubated for 45 minutes with the virion/inhibitor mixture, the cells were washed with PBS to remove free virus and then fixed for fluorescent microscopy (Figure 3 a) or harvested for flow cytometry (Figure 3 b). The results clearly show that the number of virions binding to the cells was reduced by the nano‐inhibitors with SAL functionalization. For VLNP‐SAL/Zan, there was nearly no signal for virus being detected by CLSM, and the fluorescence intensity profile for the cells was identical to the no‐virus control sample as shown in flow‐cytometry, which confirms our hypothesis that the spiky nano‐inhibitors can block virus binding to the host cells effectively.

**Figure 3:**
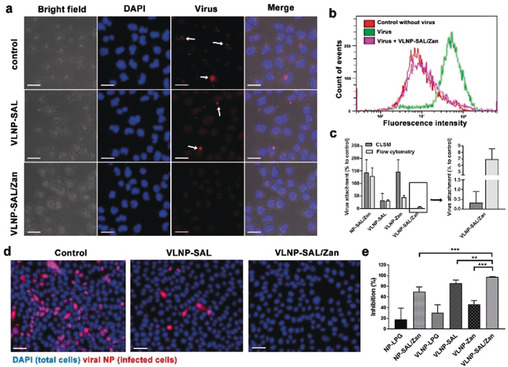
a) Projection CLSM images for virion binding to MDCK II cells in the presence of the inhibitors. Scale bar: 20 μm. b) Flow‐cytometry analysis for the virion binding to MDCK II cells in the presence of VLNP‐SAL/Zan. c) Virus attachment analysis for the CLSM images and flow‐cytometry results. Values are expressed as mean±SD, *n*=4. d) Immunofluorescence staining of the viral NP to show the cellular infections at an MOI of 1. Scale bar: 50 μm, MOI=multiplicity of infection. e) Inhibition ratios for the nano‐inhibitors from the counting of infected cells. Values are expressed as mean±SD, *n*=4. **p<0.01, ***p<0.001 by Student t‐test. Detailed data for other inhibitors are shown Figure S7 and S8.

With the proof of decoy binding, we then investigated whether the nano‐inhibitors could inhibit influenza virus infection at the entry step. Immunofluorescence staining of viral NP in the infected cells was carried out. All cells were marked with the DAPI stain (blue) while the infected cells were additionally marked by staining of the viral NP (pink) as shown in Figure 3 d,e. In control cultures, 42.2±6.6 % of the cells expressed viral antigen. Pre‐treatment with the nano‐inhibitors clearly reduced infection. Without SAL or Zan, there was no significant inhibition of viral infection, which excludes an effect by underivatized LPG for the viral inhibition (Figure S7). For the cells treated with VLNP‐SAL and VLNP‐Zan, the infection rates were 6.2±2.8 % and 22.5±2.9 %, respectively. Few to no infected cells (0.1±0.1 %) were detected upon treatment with VLNP‐SAL/Zan, which corresponds to an inhibition ratio higher than 99.9 %.

A potent influenza inhibitor should show robust inhibitory activity even when used after the infection. We evaluated inhibition of post‐infection viral replication by the nano‐inhibitors by using a multicyclic viral replication assay. In this assay, MDCK II cells were infected with virus at a multiplicity of infection (MOI) of 0.01, and then cultured in the medium supplied with the nano‐inhibitors for 24 hours. Afterwards, the active virus in the medium was titrated by plaque assay to investigate viral replication. We first investigated the inhibitory effects on influenza A/X31 (H3N2) for the inhibitors at different concentrations from 50 to 1000 μg mL^−1^ (Figure 4 a). Potent inhibition with a reduction in viral titre of six orders of magnitude, corresponding to 99.9999 % inhibition, was achieved by VLNP‐SAL/Zan at the cellular non‐toxic dose, which support a therapeutic window for the inhibitor. With only 50 μg mL^−1^ VLNP‐SAL, a reduction of 5 orders of magnitudes was achieved. NP‐SAL/Zan showed potent inhibition at high doses, but at 50 μg mL^−1^, the viral titre was reduced by only 2.5 orders of magnitudes, thus supporting the idea that the spiky nanostructures can enhance viral binding and inhibition. Similar inhibitory effects were obtained for infection with two other typical human IAV strains, A/PR/8/34 (H1N1) and A/Panama/2007/1999 (H3N2), which indicated that the VLNP‐SAL/Zan might be broadly active in human IAV strains (Figure 4 b).

**Figure 4:**
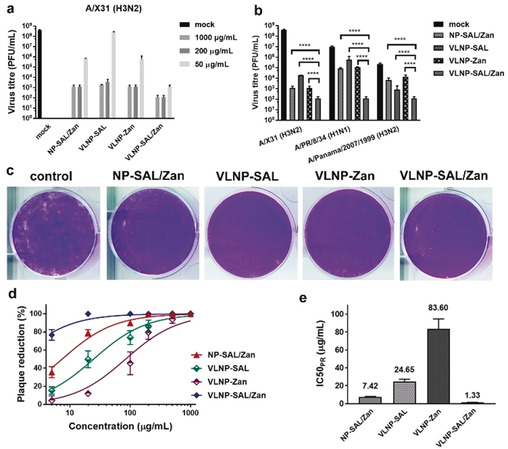
a) Inhibition of influenza A/X31 (H3N2) replication in the presence of the nano‐inhibitors. b) Inhibition of virus replication at an inhibitor dosage of 1000 μg mL^−1^. The inhibitors were introduced into the cell culture medium 45 min after the first cycle of viral infection. c) Representative images for the reduction of plaque formation of influenza A/X31 (H3N2) for inhibitors at a concentration of 100 μg mL^−1^. d) Plaque‐reduction ratios for the inhibitors at different concentrations. e) IC_50(PR)_ values for the inhibitors towards influenza A/X31 (H3N2) from the plaque‐reduction ratios. Values are expressed as mean±SD, *n*=4. ****p<0.0001 by Student t‐test.

We also evaluated inhibition of viral replication at a late stage of infection with high viral load (Figure S9). In this case, the nano‐inhibitors were used 24 hours post infection. Compared with the experimental settings for Figure 4 a,b, there were 2.5‐fold more replication cycles, with approximately 10^8^ PFU mL^−1^ virions in the medium before using the inhibitors. Reduction in viral titres was also achieved with VLNP‐SAL/Zan, for which a reduction of 5 orders of magnitudes was achieved, which was slightly lower but in the same level as its performance in the early‐stage inhibition. However, in this case, VLNP‐SAL only showed a moderate inhibition of viral replication, with a reduction of just 2 orders of magnitudes.

The potent inhibitory effects indicate that the viruses might be neutralized by the nano‐inhibitors in the medium. For verification, plaque‐reduction assays were carried out (Figure 4 c–e). Dose‐dependent plaque reduction (PR) curves were obtained by varying the nano‐inhibitor concentrations (Figure 4 d) and the IC_50_ values were then estimated (Figure 4 e). VLNP‐SAL/Zan showed the best plaque reduction performance, for which the IC_50(PR)_ is 1.33±0.14 μg mL^−1^. The tendencies for VLNP‐SAL and NP‐SAL/Zan were similar to that for VLNP‐SAL/Zan, but with higher IC_50(PR)_ values. For VLNP‐Zan, only a slight reduction in viral activity was noticed. This also indicates efficient neutralizing of the virus, for which heteromultivalent engagement with both HA and NA is essential.

In conclusion, in this study, we demonstrated the topology‐matching design of an IAV inhibitor that has a matching surface topology towards the IAV particle and shows dual inhibitory effects towards the two key proteins for viral binding. The synthesized nano‐inhibitor is able to neutralize IAV virions extracellularly and block their binding to the host cells. As a result, robust inhibition (>99.9999 % or a reductions of 6 orders of magnitude) of viral replication is achieved. We also envision that the idea of topology‐matching design could be extended to the inhibition of other viruses, especially ones with a spiky morphology such as coronaviruses.

## Conflict of interest

The authors declare no conflict of interest.

## Supporting information

As a service to our authors and readers, this journal provides supporting information supplied by the authors. Such materials are peer reviewed and may be re‐organized for online delivery, but are not copy‐edited or typeset. Technical support issues arising from supporting information (other than missing files) should be addressed to the authors.

Supplementary
